# Methyl 4-[(pyrimidin-2-yl)carbamo­yl]benzoate

**DOI:** 10.1107/S1600536811025190

**Published:** 2011-06-30

**Authors:** Chun-Hsiang Lu, Chia-Jun Wu, Chun-Wei Yeh, Hui-Ling Hu, Jhy-Der Chen

**Affiliations:** a Department of Chemistry, Chung-Yuan Christian University, Chung-Li, Taiwan; b Department of Chemical and Materials Engineering, Nanya Institute of Technology, Chung-Li, Taiwan

## Abstract

Mol­ecules of the title compound, C_13_H_11_N_3_O_3_, are connected into centrosymmetric dimers *via* inter­molecular N—H⋯N hydrogen bonds, generating an *R*_2_^2^(8) motif. The pyrimidine and the phenyl rings are twisted with respect to each other by an inter­planar angle of 61.3 (1)°.

## Related literature

For related metal complexes of the title compound, see: Wu *et al.* (2011 ▶).
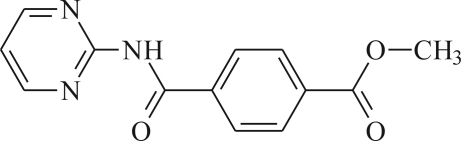


## Experimental

### 

#### Crystal data

C_13_H_11_N_3_O_3_
*M* *_r_* = 257.25
Triclinic, 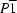

*a* = 5.7387 (7) Å
*b* = 7.9037 (10) Å
*c* = 13.6496 (19) Å
α = 80.793 (12)°
β = 79.997 (11)°
γ = 77.426 (10)°
*V* = 590.24 (13) Å^3^
*Z* = 2
Mo *K*α radiation
μ = 0.11 mm^−1^
*T* = 295 K
0.5 × 0.3 × 0.1 mm

#### Data collection

Siemens P4 diffractometer
Absorption correction: ψ scan (*XSCANS*; Siemens, 1995 ▶) *T*_min_ = 0.963, *T*_max_ = 0.989
2727 measured reflections
2066 independent reflections
1427 reflections with *I* > 2σ(*I*)
*R*_int_ = 0.037
3 standard reflections every 97 reflections  intensity decay: none

#### Refinement

*R*[*F* ^2^ > 2σ(*F* ^2^)] = 0.045
*wR*(*F* ^2^) = 0.119
*S* = 1.04
2066 reflections
174 parameters
H-atom parameters constrained
Δρ_max_ = 0.21 e Å^−3^
Δρ_min_ = −0.18 e Å^−3^

### 

Data collection: *XSCANS* (Siemens, 1995 ▶); cell refinement: *XSCANS*; data reduction: *XSCANS*; program(s) used to solve structure: *SHELXS97* (Sheldrick, 2008 ▶); program(s) used to refine structure: *SHELXL97* (Sheldrick, 2008 ▶); molecular graphics: *SHELXTL* (Sheldrick, 2008 ▶); software used to prepare material for publication: *SHELXTL*.

## Supplementary Material

Crystal structure: contains datablock(s) I, global. DOI: 10.1107/S1600536811025190/bt5561sup1.cif

Structure factors: contains datablock(s) I. DOI: 10.1107/S1600536811025190/bt5561Isup2.hkl

Supplementary material file. DOI: 10.1107/S1600536811025190/bt5561Isup3.cml

Additional supplementary materials:  crystallographic information; 3D view; checkCIF report

## Figures and Tables

**Table 1:** Hydrogen-bond geometry (Å, °)

*D*—H⋯*A*	*D*—H	H⋯*A*	*D*⋯*A*	*D*—H⋯*A*
N3—H3*B*⋯N1^i^	0.86	2.30	3.104 (3)	156

Symmetry code: (i) 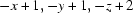.

**Fig. 1.**
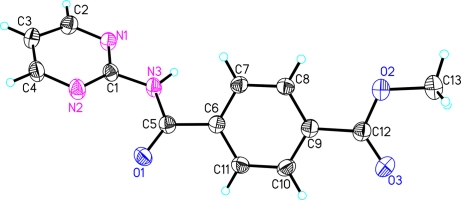
Crystal structure of the title compound with atom labeling and displacement ellipsoids drawn at the 30% probability level.

## supplementary crystallographic information

### Comment

The silver(I) complex containg methyl-4-(pyrimidin-2-ylcarbamoyl)benzoate ligand has been reported, which shows one-dimensional structure (Wu  *et al.*, 2011). Within this project the crystal structure of the title compound was determined. In its crystal structure intermolecular N—H···N hydrogen bonds are found (Tab. 1).

### Experimental

The title compound was prepared according to a published procedure (Wu *et al.*, 2011). Plate like crystals suitable for X-ray crystallography were obtained by slow evaporization of the solvent from a solution of the title compound in methanol.

### Refinement

H atoms   were placed in idealized positions and constrained to ride on their parent atoms, with C—H = 0.93 - 0.96Å and N—H = 0.86 Å, and with *U*_iso_(H) = 1.2 or 1.5 *U*_eq_(C/N).

### Crystal data

**Table:**

C_13_H_11_N_3_O_3_	*Z* = 2
*M**_r_* = 257.25	*F*(000) = 268
Triclinic, *P*1	*D*_x_ = 1.447 Mg m^−^^3^
Hall symbol: -P 1	Mo *K*α radiation, λ = 0.71073 Å
*a* = 5.7387 (7) Å	Cell parameters from 26 reflections
*b* = 7.9037 (10) Å	θ = 4.4–17.4°
*c* = 13.6496 (19) Å	µ = 0.11 mm^−^^1^
α = 80.793 (12)°	*T* = 295 K
β = 79.997 (11)°	Plate, colourless
γ = 77.426 (10)°	0.5 × 0.3 × 0.1 mm
*V* = 590.24 (13) Å^3^	

### Data collection

**Table:**

Siemens P4 diffractometer	1427 reflections with *I* > 2σ(*I*)
Radiation source: fine-focus sealed tube	*R*_int_ = 0.037
graphite	θ_max_ = 25.0°, θ_min_ = 2.7°
ω scans	*h* = −6→1
Absorption correction: ψ scan (*XSCANS*; Siemens, 1995)	*k* = −9→9
*T*_min_ = 0.963, *T*_max_ = 0.989	*l* = −16→16
2727 measured reflections	3 standard reflections every 97 reflections
2066 independent reflections	intensity decay: none

### Refinement

**Table:**

Refinement on *F*^2^	Secondary atom site location: difference Fourier map
Least-squares matrix: full	Hydrogen site location: inferred from neighbouring sites
*R*[*F*^2^ > 2σ(*F*^2^)] = 0.045	H-atom parameters constrained
*wR*(*F*^2^) = 0.119	*w* = 1/[σ^2^(*F*_o_^2^) + (0.043*P*)^2^ + 0.2307*P*] where *P* = (*F*_o_^2^ + 2*F*_c_^2^)/3
*S* = 1.04	(Δ/σ)_max_ < 0.001
2066 reflections	Δρ_max_ = 0.21 e Å^−^^3^
174 parameters	Δρ_min_ = −0.18 e Å^−^^3^
0 restraints	Extinction correction: *SHELXL97* (Sheldrick, 2008), Fc^*^=kFc[1+0.001xFc^2^λ^3^/sin(2θ)]^-1/4^
Primary atom site location: structure-invariant direct methods	Extinction coefficient: 0.035 (5)

### Special details

**Table:**

Geometry. All e.s.d.'s (except the e.s.d. in the dihedral angle between two l.s. planes) are estimated using the full covariance matrix. The cell e.s.d.'s are taken into account individually in the estimation of e.s.d.'s in distances, angles and torsion angles; correlations between e.s.d.'s in cell parameters are only used when they are defined by crystal symmetry. An approximate (isotropic) treatment of cell e.s.d.'s is used for estimating e.s.d.'s involving l.s. planes.
Refinement. Refinement of *F*^2^ against ALL reflections. The weighted *R*-factor *wR* and goodness of fit *S* are based on *F*^2^, conventional *R*-factors *R* are based on *F*, with *F* set to zero for negative *F*^2^. The threshold expression of *F*^2^ > σ(*F*^2^) is used only for calculating *R*-factors(gt) *etc*. and is not relevant to the choice of reflections for refinement. *R*-factors based on *F*^2^ are statistically about twice as large as those based on *F*, and *R*- factors based on ALL data will be even larger.

### Fractional atomic coordinates and isotropic or equivalent isotropic displacement parameters (Å^2^)

**Table:**

	*x*	*y*	*z*	*U*_iso_*/*U*_eq_	
C1	0.3108 (4)	0.2690 (3)	1.01131 (16)	0.0434 (5)	
C2	0.4585 (5)	0.1585 (3)	1.15496 (18)	0.0526 (6)	
H2A	0.5531	0.1622	1.2032	0.063*	
C3	0.3398 (5)	0.0224 (3)	1.16664 (19)	0.0556 (7)	
H3A	0.3510	−0.0647	1.2211	0.067*	
C4	0.2038 (5)	0.0214 (3)	1.09384 (19)	0.0569 (7)	
H4A	0.1188	−0.0682	1.0999	0.068*	
C5	0.1708 (4)	0.4275 (3)	0.85404 (17)	0.0441 (5)	
C6	0.2691 (4)	0.5254 (3)	0.75767 (16)	0.0410 (5)	
C7	0.5130 (4)	0.4939 (3)	0.71956 (17)	0.0448 (6)	
H7A	0.6229	0.4185	0.7570	0.054*	
C8	0.5926 (4)	0.5745 (3)	0.62600 (17)	0.0449 (6)	
H8A	0.7555	0.5503	0.6000	0.054*	
C9	0.4323 (4)	0.6908 (3)	0.57060 (16)	0.0426 (5)	
C10	0.1888 (4)	0.7234 (3)	0.60887 (18)	0.0493 (6)	
H10A	0.0797	0.8015	0.5722	0.059*	
C11	0.1086 (4)	0.6399 (3)	0.70134 (17)	0.0474 (6)	
H11A	−0.0552	0.6608	0.7261	0.057*	
C12	0.5146 (4)	0.7860 (3)	0.47167 (17)	0.0473 (6)	
C13	0.8539 (5)	0.8302 (4)	0.35163 (19)	0.0626 (7)	
H13A	1.0257	0.8119	0.3490	0.094*	
H13B	0.7869	0.9530	0.3485	0.094*	
H13C	0.8174	0.7856	0.2959	0.094*	
N1	0.4459 (3)	0.2859 (2)	1.07831 (14)	0.0469 (5)	
N2	0.1882 (4)	0.1440 (3)	1.01460 (15)	0.0558 (6)	
N3	0.3151 (4)	0.3953 (3)	0.92751 (14)	0.0496 (5)	
H3B	0.4202	0.4605	0.9213	0.059*	
O1	−0.0197 (3)	0.3799 (2)	0.86197 (13)	0.0601 (5)	
O2	0.7514 (3)	0.7402 (2)	0.44414 (12)	0.0576 (5)	
O3	0.3845 (3)	0.8936 (3)	0.42251 (14)	0.0690 (6)	

### Atomic displacement parameters (Å^2^)

**Table:**

	*U*^11^	*U*^22^	*U*^33^	*U*^12^	*U*^13^	*U*^23^
C1	0.0482 (13)	0.0487 (13)	0.0364 (12)	−0.0176 (11)	−0.0042 (10)	−0.0053 (10)
C2	0.0638 (16)	0.0542 (15)	0.0436 (13)	−0.0143 (12)	−0.0160 (12)	−0.0048 (11)
C3	0.0678 (17)	0.0504 (15)	0.0466 (14)	−0.0158 (12)	−0.0070 (12)	0.0044 (11)
C4	0.0648 (17)	0.0553 (15)	0.0551 (15)	−0.0292 (13)	−0.0085 (13)	0.0032 (12)
C5	0.0487 (14)	0.0462 (13)	0.0419 (12)	−0.0162 (11)	−0.0083 (10)	−0.0077 (10)
C6	0.0493 (14)	0.0412 (12)	0.0381 (12)	−0.0155 (10)	−0.0109 (10)	−0.0071 (9)
C7	0.0458 (13)	0.0476 (13)	0.0442 (13)	−0.0128 (10)	−0.0150 (10)	−0.0010 (10)
C8	0.0412 (13)	0.0498 (13)	0.0459 (13)	−0.0123 (10)	−0.0097 (10)	−0.0045 (11)
C9	0.0510 (14)	0.0445 (12)	0.0379 (12)	−0.0160 (10)	−0.0116 (10)	−0.0070 (10)
C10	0.0514 (14)	0.0501 (14)	0.0482 (14)	−0.0086 (11)	−0.0197 (11)	0.0001 (11)
C11	0.0422 (13)	0.0550 (14)	0.0473 (14)	−0.0130 (11)	−0.0093 (10)	−0.0051 (11)
C12	0.0557 (15)	0.0496 (14)	0.0422 (13)	−0.0179 (12)	−0.0141 (11)	−0.0046 (11)
C13	0.0716 (18)	0.0643 (17)	0.0510 (15)	−0.0245 (14)	0.0011 (13)	−0.0002 (13)
N1	0.0571 (12)	0.0483 (11)	0.0413 (11)	−0.0194 (9)	−0.0127 (9)	−0.0044 (9)
N2	0.0666 (14)	0.0570 (13)	0.0529 (13)	−0.0319 (11)	−0.0148 (10)	0.0009 (10)
N3	0.0625 (13)	0.0563 (12)	0.0405 (11)	−0.0326 (10)	−0.0164 (9)	0.0013 (9)
O1	0.0508 (10)	0.0784 (12)	0.0559 (11)	−0.0290 (9)	−0.0113 (8)	0.0042 (9)
O2	0.0591 (11)	0.0611 (11)	0.0489 (10)	−0.0155 (9)	−0.0034 (8)	0.0043 (8)
O3	0.0674 (12)	0.0794 (13)	0.0560 (11)	−0.0142 (10)	−0.0207 (9)	0.0166 (10)

### Geometric parameters (Å, °)

**Table:**

C1—N2	1.323 (3)	C7—H7A	0.9300
C1—N1	1.336 (3)	C8—C9	1.384 (3)
C1—N3	1.393 (3)	C8—H8A	0.9300
C2—N1	1.331 (3)	C9—C10	1.387 (3)
C2—C3	1.369 (4)	C9—C12	1.490 (3)
C2—H2A	0.9300	C10—C11	1.380 (3)
C3—C4	1.368 (4)	C10—H10A	0.9300
C3—H3A	0.9300	C11—H11A	0.9300
C4—N2	1.333 (3)	C12—O3	1.202 (3)
C4—H4A	0.9300	C12—O2	1.331 (3)
C5—O1	1.213 (3)	C13—O2	1.443 (3)
C5—N3	1.368 (3)	C13—H13A	0.9600
C5—C6	1.501 (3)	C13—H13B	0.9600
C6—C11	1.385 (3)	C13—H13C	0.9600
C6—C7	1.389 (3)	N3—H3B	0.8600
C7—C8	1.382 (3)		
			
N2—C1—N1	127.4 (2)	C8—C9—C10	119.4 (2)
N2—C1—N3	118.7 (2)	C8—C9—C12	121.8 (2)
N1—C1—N3	113.84 (19)	C10—C9—C12	118.8 (2)
N1—C2—C3	123.5 (2)	C11—C10—C9	120.0 (2)
N1—C2—H2A	118.3	C11—C10—H10A	120.0
C3—C2—H2A	118.3	C9—C10—H10A	120.0
C4—C3—C2	116.4 (2)	C10—C11—C6	120.8 (2)
C4—C3—H3A	121.8	C10—C11—H11A	119.6
C2—C3—H3A	121.8	C6—C11—H11A	119.6
N2—C4—C3	122.6 (2)	O3—C12—O2	123.4 (2)
N2—C4—H4A	118.7	O3—C12—C9	124.5 (2)
C3—C4—H4A	118.7	O2—C12—C9	112.1 (2)
O1—C5—N3	124.8 (2)	O2—C13—H13A	109.5
O1—C5—C6	120.6 (2)	O2—C13—H13B	109.5
N3—C5—C6	114.57 (19)	H13A—C13—H13B	109.5
C11—C6—C7	119.2 (2)	O2—C13—H13C	109.5
C11—C6—C5	118.7 (2)	H13A—C13—H13C	109.5
C7—C6—C5	121.9 (2)	H13B—C13—H13C	109.5
C8—C7—C6	120.0 (2)	C2—N1—C1	114.5 (2)
C8—C7—H7A	120.0	C1—N2—C4	115.6 (2)
C6—C7—H7A	120.0	C5—N3—C1	127.31 (19)
C7—C8—C9	120.6 (2)	C5—N3—H3B	116.3
C7—C8—H8A	119.7	C1—N3—H3B	116.3
C9—C8—H8A	119.7	C12—O2—C13	117.09 (19)

### Hydrogen-bond geometry (Å, °)

**Table:**

*D*—H···*A*	*D*—H	H···*A*	*D*···*A*	*D*—H···*A*
N3—H3B···N1^i^	0.86	2.30	3.104 (3)	156

Symmetry codes: (i) −*x*+1, −*y*+1, −*z*+2.
